# Dead or Alive? Factors Affecting the Survival of Victims during Attacks by Saltwater Crocodiles (*Crocodylus porosus*) in Australia

**DOI:** 10.1371/journal.pone.0126778

**Published:** 2015-05-11

**Authors:** Yusuke Fukuda, Charlie Manolis, Keith Saalfeld, Alain Zuur

**Affiliations:** 1 Northern Territory Department of Land Resource Management, Palmerston, Northern Territory, Australia; 2 Wildlife Management International Pty. Limited, Karama, Northern Territory, Australia; 3 Highland Statistics Limited, Newburgh, United Kingdom; University of Regina, CANADA

## Abstract

Conflicts between humans and crocodilians are a widespread conservation challenge and the number of crocodile attacks is increasing worldwide. We identified the factors that most effectively decide whether a victim is injured or killed in a crocodile attack by fitting generalized linear models to a 42-year dataset of 87 attacks (27 fatal and 60 non-fatal) by saltwater crocodiles (*Crocodylus porosus*) in Australia. The models showed that the most influential factors were the difference in body mass between crocodile and victim, and the position of victim in relation to the water at the time of an attack. In-water position (for diving, swimming, and wading) had a higher risk than on-water (boating) or on-land (fishing, and hunting near the water's edge) positions. In the in-water position a 75 kg person would have a relatively high probability of survival (0.81) if attacked by a 300 cm crocodile, but the probability becomes much lower (0.17) with a 400 cm crocodile. If attacked by a crocodile larger than 450 cm, the survival probability would be extremely low (<0.05) regardless of the victim’s size. These results indicate that the main cause of death during a crocodile attack is drowning and larger crocodiles can drag a victim more easily into deeper water. A higher risk associated with a larger crocodile in relation to victim’s size is highlighted by children’s vulnerability to fatal attacks. Since the first recently recorded fatal attack involving a child in 2006, six out of nine fatal attacks (66.7%) involved children, and the average body size of crocodiles responsible for these fatal attacks was considerably smaller (384 cm, 223 kg) than that of crocodiles that killed adults (450 cm, 324 kg) during the same period (2006–2014). These results suggest that culling programs targeting larger crocodiles may not be an effective management option to improve safety for children.

## Introduction

Conflicts between humans and wildlife, especially large carnivores such as crocodilians, are becoming a complex conservation challenge worldwide [1–3]. In the case of crocodilians, they pose a threat to people, livestock or pets in local urban and rural communities [4, 5]. Crocodile attacks result in serious injury or death of a victim in most cases [6–8], and even crocodilian species that are considered harmless to humans are often viewed with fear. During the 1950s to 1980s, many crocodilian species were threatened due to overexploitation and habitat loss [9], but protection and implementation of effective conservation programs have seen some populations achieve extensive recovery [10–12] while others remain endangered [13–15]. Conservation actions typically aim to increase depleted crocodilian populations, and the success of such conservation programs invariably leads to an increase in negative interactions between people and crocodilians (human-crocodile conflict; HCC) [16]. Increased HCC may also be related to increasing human populations [17], urbanisation and encroachment by humans into crocodile habitats for tourism, recreation, agriculture or other purposes [18].

While HCC may be an increasing issue, numerous attacks by crocodilians go unreported or are poorly documented in many countries where crocodilians are distributed [16]. Attacks by American alligators (*Alligator mississippiensis*) are well documented, although many attacks are provoked (e.g. while handling animals) [19–21]. Fatal attacks by *A*. *mississippiensis* are uncommon, reflecting their smaller size and more docile nature relative to other crocodilian species [22]. More aggressive species such as saltwater crocodiles (*Crocodylus porosus*) and Nile crocodiles (*C*. *niloticus*) [23] are responsible for a much higher mortality of humans in unprovoked attacks. Between January 2008 and October 2013, 528 attacks by *C*. *porosus* and 466 by *C*. *niloticus* were reported worldwide [24]. A lack of details for increasingly common but poorly documented attacks prevents systematic examination of the incidents, although such evidence-based information is essential for management programs to improve human safety and reduce HCC. While some statistics of crocodilian attacks are reported elsewhere [17, 21, 25, 26], there have been no studies that specifically examined factors affecting the fate of a victim, whether they would be injured or killed during an attack.

Here we examine a 42-year dataset of attacks by *C*. *porosus*, the largest [27], most aggressive [23] extant crocodilian species in northern Australia where one of the largest *C*. *porosus* populations in the world exists [28]. We hypothesize that the outcome of an attack is affected by certain factors associated with a crocodile, victim, and environment, and identify which of these factors most significantly affects the probability of a victim’s survival. We use the results to inform the key messages for public safety programs, especially for children, the sector considered most vulnerable to fatal attacks in recent years.

## Materials and Methods

We conducted this retrospective study under approval by the Northern Territory Department of Land Resource Management and the Parks and Wildlife Commission of the Northern Territory.

### Study area

**Fig 1:**
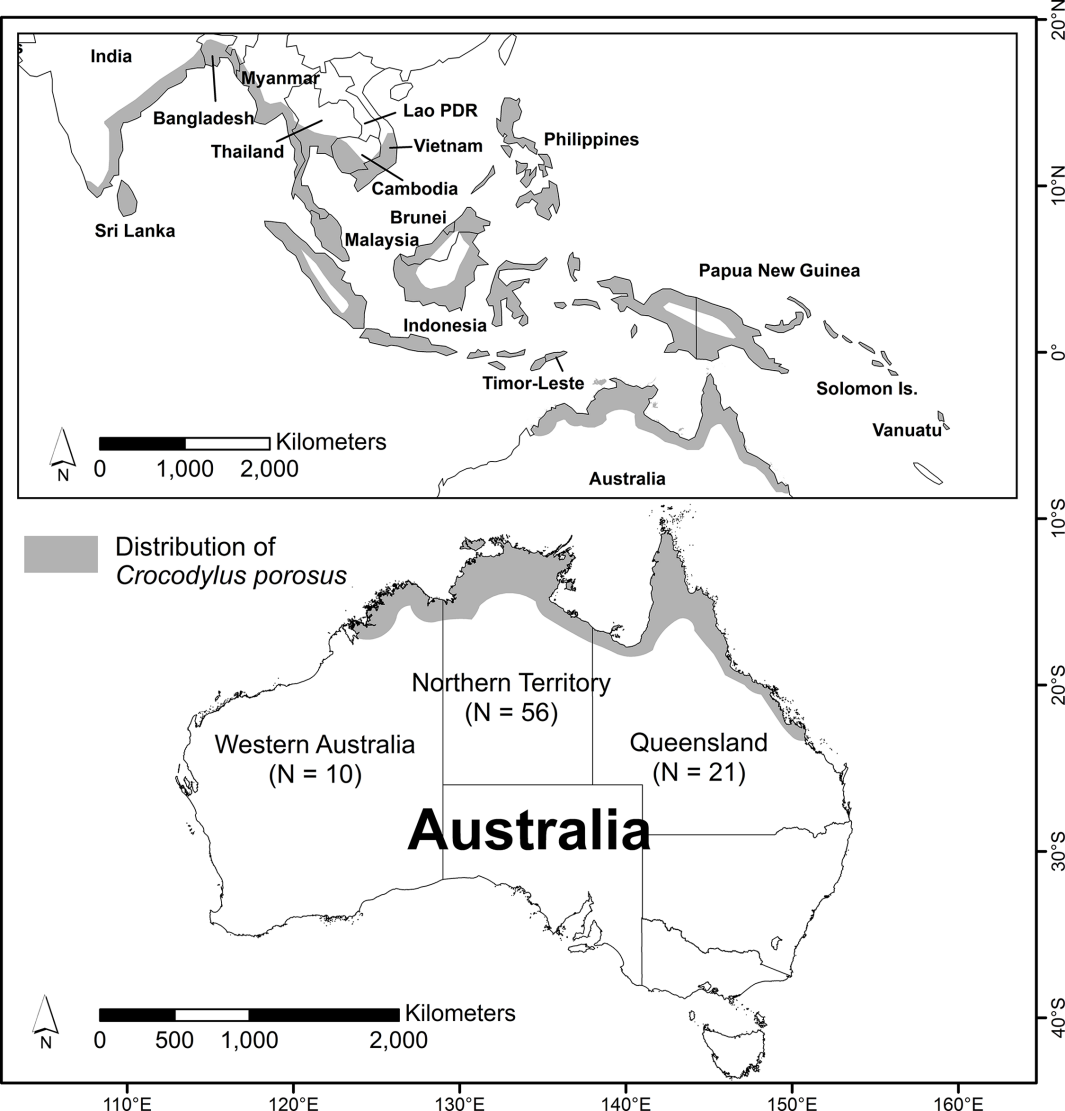
Distribution of *Crocodylus porosus* in the world and Australia. N is the number of crocodile attacks used in the analysis.

The study area was the tropical coastal regions of northern Australia, consisting of the Northern Territory, Queensland, and Western Australia. The study area covers the natural distribution of *C*. *porosus* in Australia (Fig 1) where it inhabits a range of brackish, freshwater and saline water bodies, including beaches, billabongs, floodplains, lagoons, lakes, mangroves, rivers, swamps, and waterholes [29, 30]. The climate is monsoonal with distinct wet (November-April) and dry (May-October) seasons. The annual minimum and maximum temperature typically ranges from 16 to 37°C, and the annual rainfall is 1000–1700 mm [31]. The area covers several towns and townships, including many remote indigenous communities.

### Historical data

We compiled historical records of *C*. *porosus* attacks across northern Australia since the species was protected in Western Australia (1970), the Northern Territory (1971), and Queensland (1974), by 1) collating the internal reports and databases kept within government and police agencies, 2) searching media sources, such as archived newspapers and websites, and 3) consulting with independent databases (Y. Fukuda, C. Manolis, unpublished data). We excluded: provoked attacks resulting from voluntary contact with crocodiles such as when catching crocodiles or collecting their eggs; attacks by crocodiles in captivity or escapees from crocodile farms, even if unprovoked; attacks that did not cause any injury (including death), because these attacks were often not reported; and, suspected incidents such as victims going missing without witness or evidence as a crocodile attack. In two cases two victims were involved in an attack by the same crocodile. We treated such a case as two separate attacks because our focus was whether a victim was injured or killed (result) rather than whether a victim was attacked or not (causation). We collected details of each incident, including 1) the date, time, location, and severity of an attack, 2) the total length of the crocodile either measured or estimated, and 3) the age, origin (local or visitor), and activity of the victim at the time of the incident. All of our compiled data, except for confidential information, are accessible in a publicly available database [24] and general trends are summarized by [17]. Personal information on the victims was anonymized and de-identified prior to the analyses.

### Analysis

To examine the consequence of crocodile attacks and their attributes, we modelled the relationships as a binary logistic regression, using Generalized Linear Models (GLM). We coded the crocodile attacks into fatal and non-fatal as a binary response variable (0 = fatal, 1 = non-fatal). From the information we collected for each incident, we prepared explanatory variables that we considered biologically and statistically meaningful for the management of HCC. For example, review of a few fatal cases, for which detailed information was available in coroner’s reports [32], revealed that victims were dragged under the water by a crocodile and the primary cause of their death was drowning. This indicates that the size of a crocodile was an important factor affecting the fate of a victim, but it may also be affected by the size of the victim. To account for this relationship we derived an explanatory variable, the difference in size between a crocodile and victim. We considered the size of both crocodile and human best expressed as body mass (kg) rather than length or height. Although the weight of individual crocodiles involved in incidents is unknown, we estimated it from their length, using a length-weight equation. As a previous conversion equation [30] erroneously overestimates the body mass of a crocodile, we derived a new equation by fitting an exponential function (Y = aX^b^ where Y and X are the body mass and length of a crocodile, respectively) to morphological data of saltwater crocodiles in different sizes reported in previous studies [27, 33]. We estimated the weight of victims from their sex and age, using equations obtained by fitting a quadratic function (Y = aX^2^ + bX + c where Y and X are the body mass and height of a person, respectively) to the average height and weight of people in Australia (males and females, separately) at the age of 20–80 years [34]. Because the data for the weight of people under 20 years of age were not available for Australia, we used the World Health Organization (WHO) growth reference data for 5–19 years [average height and Body Mass Index (BMI)] [35] to estimate the weight of 5–19 years old (males and females, separately). Although the WHO dataset included countries other than Australia, we assumed that these international estimates would approximate those of Australians. As a result, we derived a continuous explanatory variable, the difference in body mass between a crocodile and the victim (Δ weight).

Other explanatory variables we selected were position of a victim at the time of an incident, alcohol status of a victim (Alcohol), time of an incident (Day/night and Month), presence of a companion directly rescuing a victim from a crocodile (Assistance), age (Age), and origin (Origin) of a victim. Some of these variables were dichotomous variables such as Alcohol (intoxicated or not), Assistance (assisted or not), Day/night (day or night), Origin (local or visitor). Months were grouped into seasons (Season) that consisted of early dry (May-July), late dry (August to October), and wet (November to April) according to [36]. Age was continuous integers. Position of a victim at the time of an incident was a three-level categorical variable (on land, on water, or in water). On-land position represented fishing from the bank, hunting, and other activities such as camping near the water or collecting the water with a bucket. On-water position was boating, and in-water position included diving, swimming and wading.

We followed standard procedures for data exploration [37] and ensured that there were no outlying observations in the variables and also no collinearity between the explanatory variables. We fitted a binary logistic regression to the historical data of crocodile attacks, using the logit link function in the binomial family of GLM [38] using R version 3.1.1. We used the information-theoretic approach [39] to identify a minimum adequate model from a set of a priori models in which each model labelled M1-M10 was associated with a specific hypothesis (Table 1). Our sample size was relatively small (N = 87) and thus we used Akaike Information Criteria corrected for small samples (AICc). We compared the models using AICc and Akaike weight. We assessed the importance of each explanatory variable within the minimum adequate model, using likelihood ratio test. We then predicted the probability that a victim would survive a crocodile attack using the explanatory variables identified as most significant within the model.

**Table 1:** A priori candidate models of binary logistic regression to explain the fate of a saltwater crocodile attack.

Model	Expression	Hypothesis
M1	Δ weight + Position	Main cause of death is drowning and the risk is affected by Δ weight and Position
M2	Δ weight + Position + Alcohol	Risk of drowning is increased by the consumption of alcohol
M3	Δ weight + Position + Assistance	Risk of drowning is reduced by the assistance from the second person
M4	Season + Day/Night	Crocodiles are more active in certain seasons and day or night
M5	Alcohol + Position	Victim’s survival is reduced by their inappropriate behaviour
M6	Alcohol + Position + Age + Alcohol:Age	Alcohol consumption is more common at a certain age
M7	Sex + Age + Sex:Age	Victim’s physical strength to fight a crocodile is determined by sex and age
M8	Position + Origin + Position:Origin	Certain activities are more common for local people or visitors
M9	Position + Day/Night + Position:Day/Night	Certain activities are more common in day or night
M10	Null	None of the covariates affect victim’s survival

Based on the findings from the analysis, we provided recommendations and management implications to improve public safety and reduce the incidence of HCC, particularly in relation to a risk to children. We highlighted differences between children and adults as a victim of fatal crocodile attacks by comparing the proportion of each group since the first fatal attack on a child (2006), and the average size of crocodiles responsible for these attacks. In this study we defined children as less than 18 years of age.

## Results

**Fig 2:**
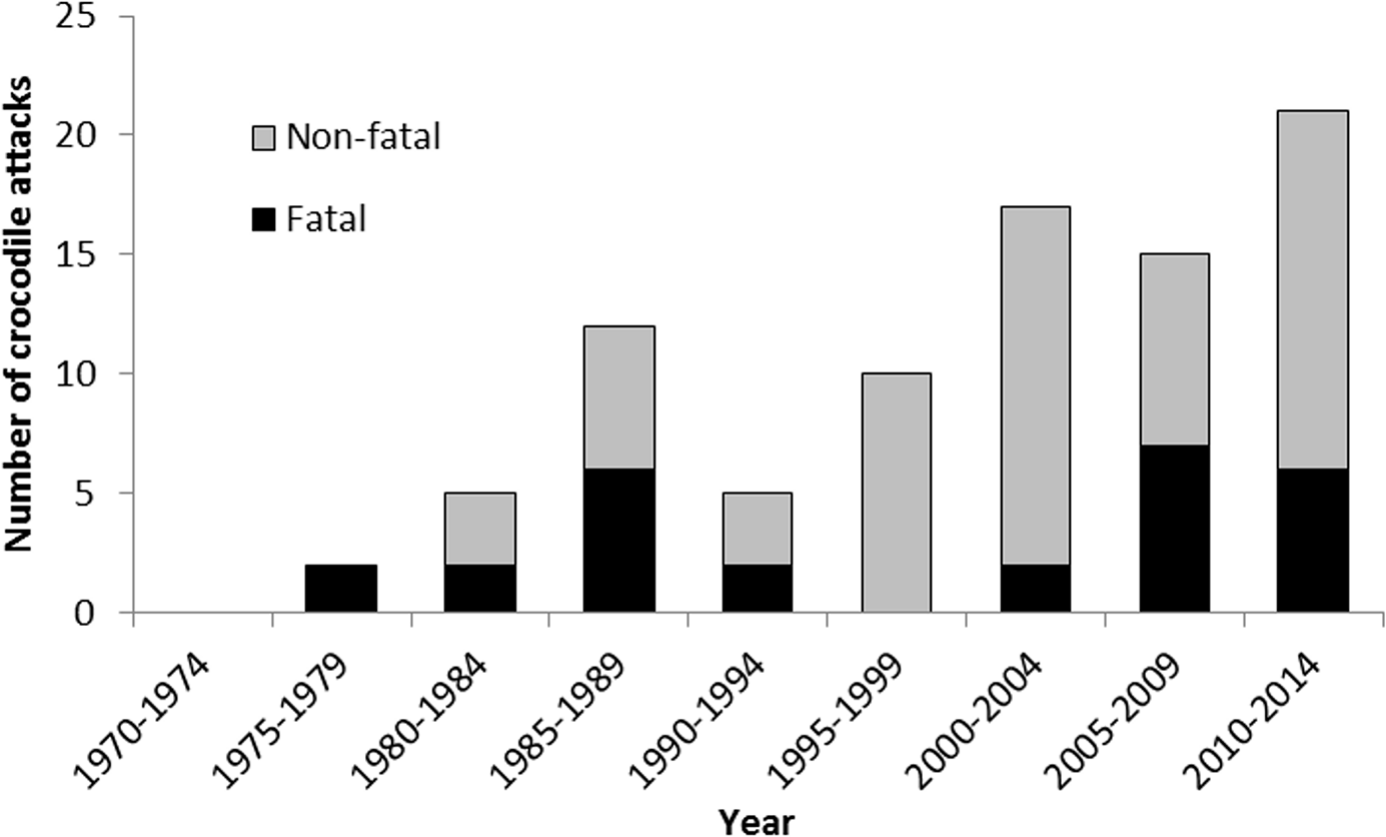
Number of fatal and non-fatal attacks by *Crocodylus porosus* divided into 5-year periods between 1970 and 2014 in Australia.

Between 1970 and 2014, there were 109 unprovoked attacks on humans by *C*. *porosus* in the wild across northern Australia. However, 22 records did not have the full detail of the crocodile, incident or victim, and were excluded from the analysis, leaving a full sample size of 87 attacks (27 fatal and 60 non-fatal). The number of attacks generally increased over years (Fig 2).

The exponential function fitted to estimate crocodile weight showed a significant fit (a = 2.552 with SE = 0.011, b = 3.321 with SE = 0.002), as did the quadratic functions fitted to estimate human weight (a_male_ = -0.012 with SE = 0.001, b_male_ = 1.201 with SE = 0.104, and c_male_ = 60.346 with SE = 2.346, and a_female_ = -0.008 with SE = 0.001, b_female_ = 0.813 with SE 0.113, and c_female_ = 52.378 with SE = 2.570). These fitted equations enabled the continuous estimation of Δ weight to be included in the candidate GLM as one of the explanatory variables.

In the set of a priori models, the smallest AICc (51.04) was achieved by M3 (Table 2). Assistance showed a large standard error in relation to the estimated coefficient (Table 3). Further investigation indicated that we had quasi-complete separation due to the Assistance variable. Quasi-complete separation is a relatively unknown feature of Bernoulli GLMs, and it occurs when a predictor (Assistance) separates the binary response variable (Survival) up to a certain high degree [40]. In this case we had the ‘Yes’ level of Assistance (N = 12) exclusively linked to ‘Non-fatal’. With quasi-complete separation, the maximum likelihood estimation of the corresponding parameter does not exist. However, the likelihood ratio test is still valid in quasi-complete separation, and results indicated that Assistance was significant (L = 3.59, df = 1, P = 0.02, Table 4).

**Table 2:** Model selection values of the candidate models of binary logistic regression.

Model	df	AICc	Model likelihood	Akaike weight
M1	4	54.15	0.21	15.88
M2	5	55.25	0.12	9.14
M3	5	51.04	>0.99	75.06
M4	4	115.14	<0.01	0.0
M5	4	99.87	<0.01	0.0
M6	6	104.0	<0.01	0.0
M7	4	114.79	<0.01	0.0
M8	6	107.69	<0.01	0.0
M9	6	105.2	<0.01	0.0
M10	1	109.82	<0.01	0.0

**Table 3:** Estimate and standard error (SE) of the explanation variables in the minimum adequate model (M3) of the binary logistic regression.

Variable	Estimate	SE
Intercept	1.91	0.56
Δ weight	-0.02	<0.01
Position (on-land)	4.91	3.55
Position (on-water)	2.58	1.38
Assistance (Yes)	18.34	2621

**Table 4:** Likelihood ratio test (LRT) of the minimum adequate model (M3) of the binary logistic regression.

Variable	Df	LRT	P
Δ weight	1	46.80	<0.01
Position	2	9.0	0.01
Assistance	1	5.36	0.02

**Fig 3:**
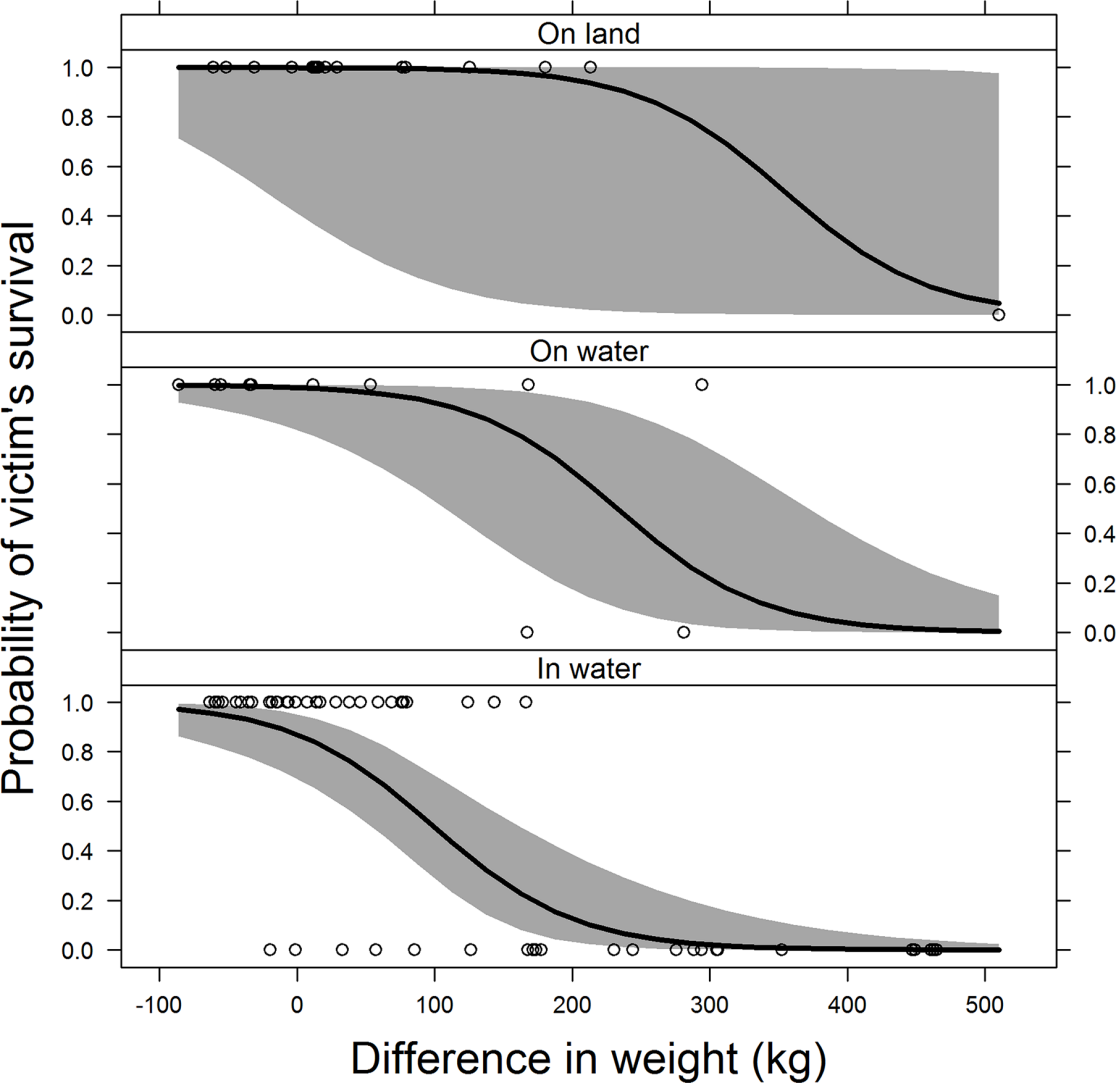
Estimated probability (solid line) that a victim survives a saltwater crocodile attack over the difference in weight between a crocodile and victim in on-land, on-water, and in-water positions. Grey shade is 95% confidence band and open symbols are the raw binary data of crocodile attacks (N = 87).

As a result we selected the model with Assistance, Δ weight, and Position (M3) as the minimum adequate model. In the selected model, Δ weight showed a much more significant effect on the response than Position while the effect of Assistance was marginal because of the quasi-complete separation (Table 4). Probability of victim’s survival decreased over the consistent range of Δ weight more rapidly in water than on water or on land (Fig 3).

The first fatal attack involving a child in Australia occurred in 2006 in the Northern Territory. Since 2006, six out of nine fatal attacks (66.7%) across Australia have involved children. The average size of crocodile involved in fatal attacks on children and adults was 384 cm (223 kg) and 450 cm (324 kg), respectively.

## Discussion

The probability of survival in a crocodile attack may not be decided by a single factor but rather a complex synergy of multiple variables in a unique situation. However, our results indicated that certain variables affected the probability of a victim’s survival more strongly than others. The difference in body mass between crocodile and victim was the most significant factor deciding the fate of a victim. In the in-water position (for diving, swimming or wading) an average-sized person weighing 75 kg would have a relatively high probability of survival (0.81) if attacked by a 300 cm crocodile. The probability of survival becomes lower (0.17) with a 400 cm crocodile, and is greatly reduced (0.02) with a 450 cm crocodile. Even a person with very large body mass (120 kg) would have a very low survival probability (0.05) with a 450 cm crocodile. This is consistent with most attacks involving >450 cm *C*. *porosus* resulting in fatalities in east India [41] and the Northern Territory of Australia [17].

The minimum size of *C*. *porosus* reported responsible for a fatal attack in Australia is 235 cm, but the victim was a 13-year-old boy whose body mass was small enough for the crocodile to drag him under the water. The probability of survival of children is lower than adults, even with a smaller crocodile. The average size of crocodiles killing children was noticeably smaller (384 cm, 223 kg) than that of crocodiles killing adults (430 cm, 324 kg). This stark contrast highlights the particular vulnerability of children to a crocodile attack. Nevertheless, it should be noted that the size of a crocodile has a much greater influence than that of a victim because crocodile body mass increases exponentially with size (e.g. a 617 cm saltwater crocodile weighed 1075 kg, [27]).

The position of a victim at the time of an incident highlights risks associated with different depth of water and its influence on the survival of the victim is significant. For a 75 kg person being attacked by a 400 cm crocodile, on-water (boating) and on-land (fishing, hunting or other) positions improves the probability of their survival from 0.17 to 0.73 and 0.97, respectively. There were only two fatal cases associated with boating, although boating is a common leisure activity in the study area. It should be noted that the victims of both fatal cases were in small (<350 cm long) boats, including a canoe. The use of boats smaller than 450 cm in habitats containing *C*. *porosus* carries a higher risk. If attacked by a 450 cm crocodile, the probability of survival of a victim on water lowers to 0.21, but that of a victim on land remains relatively high (0.73).

Taking together the effect of the body mass and position, our results suggest that the primary cause of death during a crocodile attack is drowning. This is further supported by observations in the Northern Territory that the bodies of some of the victims retrieved within 24 hours after a fatal attack were relatively intact with no major trauma [32] (Dani Best unpublished data). Drowning is also reported as a major cause of death in attacks by *A*. *mississippiensis* [20] and *C*. *porosus* elsewhere [41]. This contrasts with shark attacks where most victims die of excessive loss of blood [42]. In none of the fatal post-1970 crocodile attacks was there evidence that the victim escaped the attack and died as a result of wounds later. However, in four attacks by *C*. *porosus* in Australia before 1873, the victim reportedly escaped from the crocodile, but died later as a result of the wounds received (Charlie Manolis unpublished data). These historical cases are considered to reflect the lack of medical facilities and transport at that time. Improved communication, access to remote locations and medical care has most likely prevented some non-fatal attacks resulting in fatality due to trauma.

Another variable potentially important as shown by the likelihood ratio test was the presence of a second person proving assistance to a victim. All attacks in Australia by crocodiles larger than 400 cm on un-aided victims in the water were fatal [22], but the presence of company increased the survival probability of a victim in most cases by preventing them from being dragged into the water and moving them out of range.

These factors identified as significant provide important implications for the mitigation of HCC. Safety awareness programs need to be designed to target the risk to children from crocodile attack. In five of the six fatal cases involving children, the victim was swimming in deep water, which is the highest risk activity that a person may undertake in crocodile habitat. Given that any waterway within the range of *C*. *porosus* in northern Australia should be considered occupied habitat due to the recovery of crocodile populations to near pre-unrestricted hunting levels [12, 30], any swimming activity other than in locations identified “safe” by the authority poses an unacceptable risk. More crocodile attacks tend to happen at the beginning and end of the wet season although fatal cases occur all year around [17]. However, the survival of a victim does not depend on seasons or time of a day (daylight or night). This suggests that extreme caution should be exercised whenever entering crocodile habitats.

Inevitably, following any attack there are calls for culling of the crocodile population to reduce the risk of crocodile attack [43]. If culling were considered a management option, it would most likely target larger animals as these are commonly seen by the public as posing the greatest risk. Intensive culling could reduce the encounter rate with larger individuals. However, the effect of removing dominant individuals on the dynamics of a population is unknown [44]. Removal of large crocodiles may lead to a higher number of subordinate individuals. It should be noted that children would remain vulnerable to smaller crocodiles even if larger crocodiles are removed. To assure safety, any culling program would have to remove all crocodiles from a location, which is not a practical option for management given the high mobility and dispersal of the species across a range of habitats [45–47]. Culling programs would not ensure the absence of crocodiles in a targeted area [43, 48] and water-related activities in crocodile habitats would remain unsafe to the public. Within a decade after protection four fatal attacks occurred even though crocodile abundance was very low across northern Australia. Continuous public education campaigns to raise the safety awareness may be a more effective management option to reduce HCC. In many cases, crocodiles attacks can be prevented through such education programs [17].

Crocodile attacks are not a major cause of mortality in Australia. In the study area between 2004 and 2013, 0.02 deaths per 100,000 people (12 in total) were associated with crocodile attacks, in contrast to 8.12 deaths per 100,000 (5432) by road accidents [49]. However, the frequency of crocodile attacks has been increasing [17, 22] and management programs should incorporate evidence-based options to mitigate HCC. Our findings and recommendations may apply to other countries where *C*. *porosus* is distributed, as well as to other species known to commonly attack people [16] such as American crocodile (*C*. *acutus*), Mugger crocodile (*C*. *palustris*) and *C*. *niloticus*.

## References

[1] Treves A, Karanth KU. Human-carnivore conflict and perspectives on carnivore management worldwide. Conserv Biol. 2003;17: 1491–1499. 10.1111/j.1523-1739.2003.00059.x

[2] Treves A, Wallace RB, Naughton-Treves L, Morales A. Co-managing human–wildlife conflicts: a review. Hum Dimens Wildl. 2006;11: 383–396. 10.1080/10871200600984265

[3] Dickman AJ. Complexities of conflict: the importance of considering social factors for effectively resolving human-wildlife conflict. Anim Conserv. 2010;13: 458–466. 10.1111/j.1469-1795.2010.00368.x

[4] Aust P, Boyle B, Fergusson R, Coulson T. The impact of Nile crocodiles on rural livelihoods in northeastern Namibia. South Afr J Wildl Res. 2009;39: 57–69. 10.3957/056.039.0107

[5] Gopi GV, Pandav B. Humans sharing space with *Crocodylus porosus* in Bhitarkanika Wildlife Sanctuary: conflicts and options. Curr Sci. 2009;96: 459–460.

[6] Mekisic AP, Wardill JR. Crocodile attacks in the Northern Territory of Australia. Med J Aust. 1992;157: 751–754. 1453999 10.5694/j.1326-5377.1992.tb141275.x

[7] Gruen RL. Crocodile attacks in Australia: challenges for injury prevention and trauma care. World J Surg. 2009;33: 1554–1561. 10.1007/s00268-009-0103-6 19543941

[8] Wamisho BL, Bates J, Tompkins M, Islam R, Nyamulani N, Ngulube C, et al Ward round—crocodile bites in Malawi: microbiology and surgical management. Malawi Med J. 2009;21: 29–31. 10.4314/mmj.v21i1.10986 19780476 PMC3345715

[9] Martin S. Global diversity of crocodiles (Crocodilia, Reptilia) in freshwater. Hydrobiologia. 2008;595: 587–591. 10.1007/s10750-007-9030-4

[10] Platt SG, Thorbjarnarson JB. Population status and conservation of Morelet’s crocodile, *Crocodylus moreletii*, in northern Belize. Biol Conserv. 2000;96: 21–29. 10.1016/S0006-3207(00)00039-2

[11] Mazzotti FJ, Brandt LA, Moler P, Cherkiss MS. American crocodile (*Crocodylus acutus*) in Florida: recommendations for endangered species recovery and ecosystem restoration. J Herpetol. 2007;41: 122–132. 10.1670/0022-1511(2007)41[122:ACCAIF]2.0.CO;2

[12] Fukuda Y, Webb G, Manolis C, Delaney R, Letnic M, Lindner G, et al Recovery of saltwater crocodiles following unregulated hunting in tidal rivers of the Northern Territory, Australia. J Wildl Manag. 2011;75: 1253–1266. 10.1002/jwmg.191

[13] Thorbjarnarson J, Wang X, Ming S, He L, Ding Y, Wu Y, et al Wild populations of the Chinese alligator approach extinction. Biol Conserv. 2002;103: 93–102. 10.1016/S0006-3207(01)00128-8

[14] Thapaliya BP, Khadka M, Kafley H. Population Status and Distribution of Gharial (*Gavialis gangeticus*) in Nepal. The Initiation. 2010;3 10.3126/init.v3i0.2422

[15] Van Weerd M. Philippine Crocodile *Crocodylus mindorensis* In: Manolis SC, Stevenson C, editors. Crocodiles. Status Survey and Conservation Action Plan. Third Edition Darwin, Australia: Crocodile Specialist Group; 2010 pp. 71–78. Available: http://www.iucncsg.org/365_docs/attachments/protarea/13_C-511712ed.pdf.

[16] Sideleau B, Britton ARC. A preliminary analysis of worldwide crocodilian attacks. Crocodiles Proceedings of the 21st Working Meeting of the IUCN-SSC Crocodile Specialist Group. Gland, Switzerland: IUCN; 2012. pp. 111–114.

[17] Fukuda Y, Manolis C, Appel K. Management of human-crocodile conflict in the Northern Territory, Australia: review of crocodile attacks and removal of problem crocodiles. J Wildl Manag. 2014;78: 1239–1249.

[18] Elsey RM, Woodward AR. American alligator *Alligator mississippiensis* In: Manolis SC, Stevenson C, editors. Crocodiles. Status Survey and Conservation Action Plan. Darwin, Australia: Crocodile Specialist Group; 2010 pp. 1–4. Available: http://www.iucncsg.org/365_docs/attachments/protarea/01_A-81db765a.pdf.

[19] Conover MR, Dubow TJ. Alligator attacks on humans in the United States. Herpetol Rev. 1997;28: 120–124.

[20] Harding BE, Wolf BC. Alligator attacks in southwest Florida. J Forensic Sci. 2006;51: 674–677. 10.1111/j.1556-4029.2006.00135.x 16696720

[21] Langley RL. Adverse encounters with alligators in the United States: an update. Wilderness Environ Med. 2010;21: 156–163. 10.1016/j.wem.2010.02.002 20591380

[22] Manolis SC, Webb GJW. Assessment of saltwater crocodile (*Crocodylus porosus*) attacks in Australia (1971–2013): implications for management. Crocodiles Proceedings of the 22nd Working Meeting of the IUCN-SSC Crocodile Specialist Group. Gland, Switzerland: IUCN; 2013. pp. 97–104.

[23] Brien ML, Lang JW, Webb GJ, Stevenson C, Christian KA. The good, the bad, and the ugly: agonistic behaviour in juvenile crocodilians. PLoS ONE. 2013;8: e80872 10.1371/journal.pone.0080872 24349018 PMC3859503

[24] Charles Darwin University, Big Gecko. CrocBITE Worldwide Crocodilian Attack Database [Internet]. 2014 [cited 17 Jan 2014]. Available: http://www.crocodile-attack.info/.

[25] Caldicott DGE, Croser D, Manolis C, Webb G, Britton A. Crocodile attack in Australia: an analysis of its incidence and review of the pathology and management of crocodilian attacks in general. Wilderness Environ Med. 2005;16: 143–159. 16209470 10.1580/1080-6032(2005)16[143:CAIAAA]2.0.CO;2

[26] Dunham KM, Ghiurghi A, Cumbi R, Urbano F. Human–wildlife conflict in Mozambique: a national perspective, with emphasis on wildlife attacks on humans. Oryx. 2010;44: 185–193. 10.1017/S003060530999086X

[27] Britton ARC, Whitaker R, Whitaker N. Here be a dragon: exceptional size in a saltwater crocodile (*Crocodylus porosus*) from the Philippines. Herpetol Rev. 2012;43: 541–546.

[28] Webb GJW, Manolis SC, Brien ML. Saltwater Crocodile *Crocodylus porosus* In: Manolis SC, Stevenson C, editors. Crocodiles. Status Survey and Conservation Action Plan. Third Edition Darwin, Australia: Crocodile Specialist Group; 2010 pp. 99–113. Available: http://www.iucncsg.org/365_docs/attachments/protarea/18%20—8088e67a.pdf.

[29] Webb G, Manolis SC. Crocodiles of Australia. Sydney, Australia: Reed Books; 1989.

[30] Fukuda Y, Whitehead P, Boggs G. Broad-scale environmental influences on the abundance of saltwater crocodiles (*Crocodylus porosus*) in Australia. Wildl Res. 2007;34: 167–176.

[31] Bureau of Meteorology. Climate Data Online [Internet]. 2014 [cited 17 Jan 2014]. Available: http://www.bom.gov.au/climate/data/index.shtml.

[32] Cavanagh G. Inquest into the death of Isobel Von Jordan [2004] NTMC 09 [Internet]. Coroner’s Court Darwin; 2004. Available: http://www.nt.gov.au/justice/courtsupp/coroner/findings/2004/von_jordan.pdf.

[33] Webb GJW, Messel H. Morphometric analysis of *Crocodylus porosus* from the north coast of Arnhem Land, northern Australia. Aust J Zool. 1978;26: 1–27.

[34] Australian Bureau of Statistics. 4364.0.55.001—Australian Health Survey: First Results, 2011–12 [Internet]. 2012 [cited 20 Jan 2014]. Available: http://www.abs.gov.au/AUSSTATS/abs@.nsf/DetailsPage/4364.0.55.0012011-12?OpenDocument.

[35] World Health Organization. Growth reference data for 5–19 years [Internet]. 2006 [cited 20 Jan 2014]. Available: http://www.who.int/growthref/en/.

[36] Webb GJW. The influence of season on Australian crocodiles In: Ridpath MG, Haynes CD, Williams MJD, editors. Monsoonal Australia—Landscape, Ecology and Man in the Northern Lowlands. Rotterdam, Netherlands: A.A. Balkema; 1991 pp. 125–131.

[37] Zuur AF, Ieno EN, Elphick CS. A protocol for data exploration to avoid common statistical problems. Methods Ecol Evol. 2010;1: 3–14. 10.1111/j.2041-210X.2009.00001.x

[38] Crawley MJ. Statistics: An Introduction using R. West Sussex, England: John Wiley & Sons, Inc.; 2005.

[39] Burnham KP, Anderson DR. Model Selection and Multimodel Inference: a Practical Information-Theoretic Approach. Second edition New York, NY: Springer-Verlag; 2002.

[40] Allison PD. Convergence Problems in Logistic Regression In: Altman M, Gill J, McDonald MP, editors. Numerical Issues in Statistical Computing for the Social Scientist. Hoboken, NJ: John Wiley & Sons; 2004 pp. 238–252.

[41] Kar SK, Bustard HR. Saltwater crocodile attacks on man. Biol Conserv. 1983;25: 377–382. 10.1016/0006-3207(83)90071-X

[42] Woolgar JD, Cliff G, Nair R, Hafez H, Robbs JV. Shark Attack: Review of 86 Consecutive Cases. J Trauma Acute Care Surg. 2001;50 Available: http://journals.lww.com/jtrauma/Fulltext/2001/05000/Shark_Attack__Review_of_86_Consecutive_Cases.19.aspx. 10.1097/00005373-200105000-00019 11371847

[43] Webb G. Crocodile culls won’t solve crocodile attacks. In: The Conversation [Internet]. 2012 [cited 13 Feb 2014]. Available: http://theconversation.com/crocodile-culls-wont-solve-crocodile-attacks-11203.

[44] Campbell H, Dwyer R. Controlling crocs means knowing who’s boss. In: The Conversation [Internet]. 2013 [cited 14 Jan 2015]. Available: http://theconversation.com/controlling-crocs-means-knowing-whos-boss-13854.

[45] Brien ML, Read MA, McCallum HI, Grigg GC. Home range and movements of radio-tracked estuarine crocodiles (*Crocodylus porosus*) within a non-tidal waterhole. Wildl Res. 2008;35: 140–149.

[46] Campbell HA, Watts ME, Sullivan S, Read MA, Choukroun S, Irwin SR, et al Estuarine crocodiles ride surface currents to facilitate long-distance travel. J Anim Ecol. 2010;79: 955–964. 10.1111/j.1365-2656.2010.01709.x 20546063

[47] Campbell HA, Dwyer RG, Irwin TR, Franklin CE. Home range utilisation and long-range movement of estuarine crocodiles during the breeding and nesting season. PLOS ONE. 2013;8: e62127 10.1371/journal.pone.0062127 23650510 PMC3641080

[48] Britton A, Campbell A. Open season on crocodiles is not the solution to attacks on people. In: The Conversation [Internet]. 2014 [cited 1 Sep 2014]. Available: http://theconversation.com/open-season-on-crocodiles-is-not-the-solution-to-attacks-on-people-30722.

[49] Department of Infrastructure and Regional Development, Australian Government. Australian Road Deaths Database [Internet]. 2014 [cited 8 May 2014]. Available: http://www.bitre.gov.au/statistics/safety/fatal_road_crash_database.aspx.

[50] Leach G, Delaney R, Fukuda Y. Management Program for the Saltwater Crocodile in the Northern Territory of Australia, 2009–2014. Darwin, Australia: Northern Territory Department of Natural Resources, Environment, the Arts and Sport; 2009.

[51] Northern Territory of Australia. Animal Welfare Act [Internet]. 2013 [cited 17 Jan 2014]. Available: http://notes.nt.gov.au/dcm/legislat/legislat.nsf/d989974724db65b1482561cf0017cbd2/28ae66acac5f957569257bd7000a75f2?OpenDocument.

[52] Natural Resource Management Ministerial Council (NRMMC). Code of Practice for the Humane Treatment of Wild and Farmed Australian Crocodiles [Internet]. 2009 [cited 17 Jan 2014]. Available: http://www.environment.gov.au/resource/code-practice-humane-treatment-wild-and-farmed-australian-crocodiles.

